# Neuroplasticity-dependent and -independent mechanisms of chronic deep brain stimulation in stressed rats

**DOI:** 10.1038/tp.2015.166

**Published:** 2015-11-03

**Authors:** F R Bambico, T Bregman, M Diwan, J Li, S Darvish-Ghane, Z Li, B Laver, B O Amorim, L Covolan, J N Nobrega, C Hamani

**Affiliations:** 1 Research Imaging Centre, Centre for Addiction and Mental Health, Toronto, ON, Canada; 2 Disciplina de Neurofisiologia, Universidade Federal de São Paulo, São Paulo, Brazil; 3 Campbell Family Mental Health Research Institute, Centre for Addiction and Mental Health, Toronto, ON, Canada; 4 Division of Neurosurgery, Toronto Western Hospital, University of Toronto, Toronto, ON, Canada

## Abstract

Chronic ventromedial prefrontal cortex (vmPFC) deep brain stimulation (DBS) improves depressive-like behaviour in rats via serotonergic and neurotrophic-related mechanisms. We hypothesise that, in addition to these substrates, DBS-induced increases in hippocampal neurogenesis may also be involved. Our results show that stress-induced behavioural deficits in the sucrose preference test, forced swim test, novelty-suppressed feeding test (NSFT) and elevated plus maze were countered by chronic vmPFC DBS. In addition, stressed rats receiving stimulation had significant increases in hippocampal neurogenesis, PFC and hippocampal brain-derived neurotrophic factor levels. To block neurogenesis, stressed animals given DBS were injected with temozolomide. Such treatment reversed the anxiolytic-like effect of stimulation in the NSFT without significantly affecting performance in other behavioural tests. Taken together, our findings suggest that neuroplastic changes, including neurogenesis, may be involved in specific anxiolytic effects of DBS without affecting its general antidepressant-like response.

## Introduction

The pathophysiology of depressive disorders is complex and not adequately understood. At present, there is substantial evidence implicating neurogenic processes and neurotrophic regulation in key limbic structures of the depressive brain.^1, 2, 3, 4^ The timeframe for the therapeutic effects of antidepressants, particularly selective serotonin reuptake inhibitors, coincides with that required for the maturation of nascent granule cells in the dentate gyrus.^1, 4, 5^ Disrupting neurogenesis (for example, via irradiation) blocks the behavioural effects of serotonin reuptake inhibitors in animal models.^1, 3^ In addition to hippocampal neurogenesis, antidepressants upregulate brain-derived neurotrophic factor (BDNF) in various brain regions.^2, 6^

In the clinic, subgenual cingulate region deep brain stimulation (DBS) is being investigated for the treatment of depression.^7, 8, 9^ In a series of preclinical studies, we found that DBS delivered to the rodent homologue of the human subgenual cingulate region (that is, the ventromedial prefrontal cortex (vmPFC)) induces antidepressant- and anti-anhedonic-like responses.^10, 11, 12, 13, 14^ The therapeutic mechanisms of this effect appear to be complex. At commonly used stimulation parameters (for example, high frequencies in the order of 100 Hz), DBS has been shown to induce a functional inhibition of neuronal populations while exciting axons and fibre pathways near the target.^15, 16, 17^ Through the latter, vmPFC stimulation has been suggested to increase serotonin and BDNF levels at a distance from the target. Recent studies in rodents have shown that stimulation of limbic structures (for example, the entorhinal cortex and the anterior thalamic nucleus)^18, 19, 20, 21^ and the nucleus accumbens increases hippocampal neurogenesis.^22^ To date, the effects of vmPFC DBS on neurogenesis have only been addressed in the context of memory.^23^

The objective of the present study was to determine the role hippocampal neurogenesis on the antidepressant-, antianhedonic- and antianxiety-like effects of vmPFC stimulation. We were particularly interested in distinguishing neurogenesis-dependent and -independent DBS effects. To address this question, animals exposed to chronic unpredictable mild stress (CUS) received chronic vmPFC stimulation, followed by a battery of behavioural tests to measure depressive- and anxiety-like behaviour. Similar experiments were conducted in animals given temozolomide (TMZ), a chemotherapeutic agent known to block hippocampal neurogenesis.^24^ A second point-of-interest was to examine the role of BDNF on the effects of DBS.

## Materials and methods

Procedures were approved by the Animal Care committee of the Centre for Addiction and Mental Health. Male Fisher rats (200 g) were used in all experiments. Animals were housed in a normal light/dark cycle and tested during the day. The timeline of our experiments is summarised in Supplementary Figure 1.

### SPT and CUS

One week after arrival in the animal facility, rats were subjected for 3 days to a 1 h per day exposure to a 1% sucrose solution. This was followed by 22–23 h of water deprivation and food restriction (30 g per animal). Animals were then allowed to choose between sucrose and plain water bottles for 1 h. The sucrose preference index (SPI=sucrose intake/total fluid intake × 100) was calculated weekly and taken as a measurement of a hedonic-like state. Based on SPI scores, animals were paired and assigned to non-stressed or stressed groups. The latter was exposed to CUS until anhedonia-like responses (for example, reduction in SPI) were overtly stable (~4 weeks). Details of the stress regimen are provided in Supplementary Table 1.^25^

### Surgical procedures and electrical stimulation

After the fourth week of CUS (week 5 after baseline measurements), animals were anaesthetised with isoflurane and bilaterally implanted with insulated stainless-steel electrodes into the vmPFC (cathodes; AP+3.0, L±0.4, and V5.6 mm).^26^ Anodes were electrodes wrapped around screws implanted over the sensorimotor cortex.^12^ Controls had holes drilled into the skull but were not implanted with electrodes.^12^ DBS was conducted for 3 consecutive weeks (MTS stimulator; St Jude Medical, Plano, TX, USA) from the week after electrode implantation. The following settings were used: 100 μA, 130 Hz, 90 μs, 8 h per day, 7 days per week.^12^

### BrdU and TMZ

BrdU (5-bromo-2′-deoxyuridine; Sigma) was injected from stimulation days 7–10 (50 mg kg^−1^ twice per day). This timeline is similar to that used in studies investigating chronic effects of antidepressant medications on hippocampal neurogenesis.^1, 4, 27^ To block hippocampal neurogenesis, TMZ was administered prior to BrdU injections from stimulation days 3–5 (50 mg kg^−1^ per day).^21^ This timeframe was based on previous reports^21^ and chosen so that TMZ could affect DBS-induced BrdU+ cells that would be mature and ready to be recruited during behavioural tests (for example, 3–5 weeks later).^28^

### Behavioural tests

Behavioural tests begun 4 weeks after electrode implantation (3 days after DBS offset) and took place over a 10-day-period in the following order: novelty-suppressed feeding (NSFT) on day 3, open field (OFT) on day 5, elevated plus maze (EPMT) on day 6 and forced swim test (FST) on days 8 and 9. This sequence was chosen so that animals were subjected to the more stressful paradigms at the end. In this study, we opted not to counterbalance the test order, as a large number of animals would have been required for a clear interpretation of behavioural findings. As in previous reports,^12^ we chose not to deliver DBS during behavioural tests to avoid the confounding factor of stimulation on performance. In addition, as neurogenesis and an increase in BDNF are plastic events, we have reasoned that these lasting processes would outlast DBS offset.

#### NSFT

Rats were initially food deprived for 48 h. During test day, they were placed in a chamber containing 12 chow pellets and the latency to initiate feeding was noted.

#### OFT

Ambulatory activity while exploring an open chamber (total distance travelled) was recorded during 5 min.

#### EPMT

Rats were allowed to explore the maze for 5 min, during which the frequency and time spent in the open and closed arms were assessed.

#### FST

Rats were immersed in a cylinder filled with water for 15 min on day 1 and 5 min on day 2. During the latter session, immobility, swimming and climbing were timed.

While the FST is considered to be a screener of antidepressant-like therapies, the remainder tests assess anxiety-like behaviour. All are based on the fact that rats tend to explore novel environments. The OFT measures locomotor activity in an open arena. In the NSFT, the animals have to explore a novel environment to obtain a food reward in the middle of the apparatus. In the EPM, animals often prefer to remain in the closed arms of a maze placed at a certain height from the floor. An anxiolytic response is annotated when animals explore the open arms.

### BDNF measurements

Unperfused brains were removed, frozen over dry ice and stored at −80 °C. BDNF protein in the mPFC (prelimbic and infralimbic subregions) and hippocampus was quantified using an ELISA kit (Promega, Madison, WI, USA), according to manufacturer's instructions.

### Histology and hippocampal neurogenesis

Animals were anaesthetised with ketamine/xylazine and transcardially perfused with PBS, followed by 4% paraformaldehyde. Brains were removed from the skull and post-fixed overnight. Free-floating 40-μm sections were cut on a cryostat and processed as previously described.^19^ The following antibodies were used: primary- rat anti-BrdU (1:200; Axyl, Westbury, NY, USA), mouse anti-NeuN (1:1000, Millipore, Ontario, Canada), secondary-goat anti-rat Alexa Fluor 488 (1:200; Life Technology, Thermo Fisher Scientific, Ontario, Canada) and goat anti-mouse Rhodamine Red X (1:200; Jackson Lab, West Grove, PA, USA).

Cell counting was performed in the dentate gyrus granule cell layer and the 50-μm border along the hilar margin. Stained BrdU+ nuclei were scored in every sixth section throughout the rostrocaudal extent of the granule cell layer. All BrdU+ cells were counted bilaterally in each of the sections using a Nikon Eclipse E600 microscope and the average values for each animal were considered. For confocal microscopy, an Olympus Fluoview FV1200 microscope was used. In this study, we did not investigate subventricular neurogenesis, as this has not been affected by DBS in previous work.^21^

Location of electrode tracks was confirmed with cresyl violet staining. Animals with misplaced electrodes or lost caps were excluded from the study.

### Statistical analyses

Initial behavioural and brain measures were analysed with two-way analysis of variance (ANOVA; least significant difference *post hoc*; DBS and stress as factors). Sequential sucrose preference data were analysed with repeated measures ANOVA (time and treatment groups as factors). In stressed and non-stressed animals, the effects of TMZ were assessed using two-way ANOVA (DBS and TMZ as factors). The relationship between neurochemical and behavioural scores was carried out with Pearson correlations and regression analyses. Percentage scores (comparisons between Stress, Stress+DBS and Stress+DBS+TMZ) were calculated as ((1−Stress DBS or Stress DBS TMZ/Stress) × 100). Sample size was estimated based on the efficacy of DBS in our previous behavioural studies using the CUS model.^12^ Different batches of rats were used during different experiments. Animals perfused for histochemical analysis or non-perfused for measurement of BDNF levels were selected at random. Investigators blinded to the animals' identification conducted behavioural scoring and neurochemical analyses.

## Results

As shown in Table 1, two-way ANOVA revealed significant interactions between DBS and stress in the sucrose preference test (SPT), NSFT, FST and EPM. In contrast, no DBS or Stress effects were found in the OFT.

### Chronic DBS induces anxiolytic-, antidepressant- and antianhedonic-like effects in stressed rats

#### Stress effect

**Figure 1:**
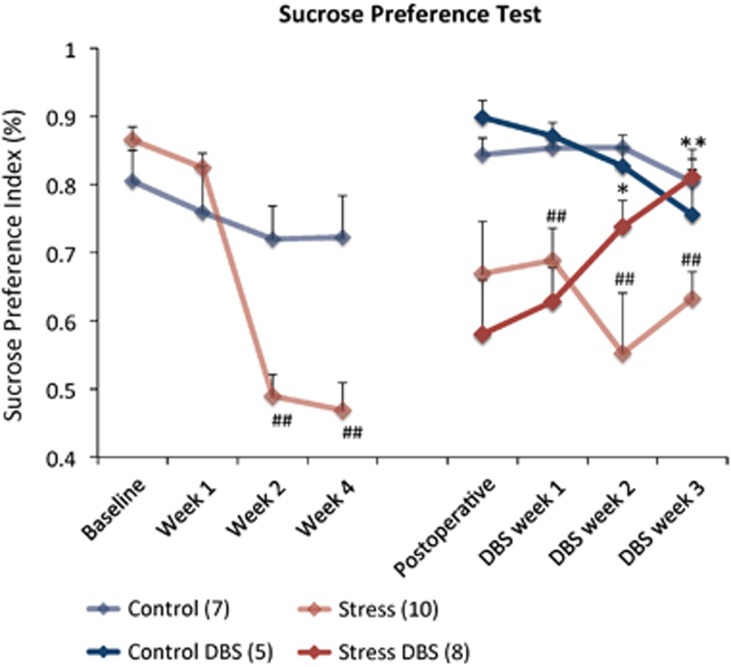
Response to DBS in the sucrose preference test. After baseline values were obtained, animals underwent 4 weeks of chronic unpredictable stress (CUS; week 3 was omitted for clarity). The post-operative measure was collected prior to DBS onset. vmPFC stimulation was delivered for 3 weeks from the second post-operative week. After surgery, a significant effect of treatment was found. On the second week of stimulation, sucrose preference in DBS-treated animals was significantly higher than in non-stimulated CUS rats. On DBS week 3, levels were similar to those recorded in non-stressed controls. ^##^*P*<0.01 stressed rats compared with non-stressed controls. *P=0.01, **P=0.002 stressed rats receiving DBS compared with non-stimulated stressed animals. Values are means and s.e. Group sizes are indicated in parenthesis. DBS, deep brain stimulation; vmPFC, ventromedial prefrontal cortex.

**Figure 2:**
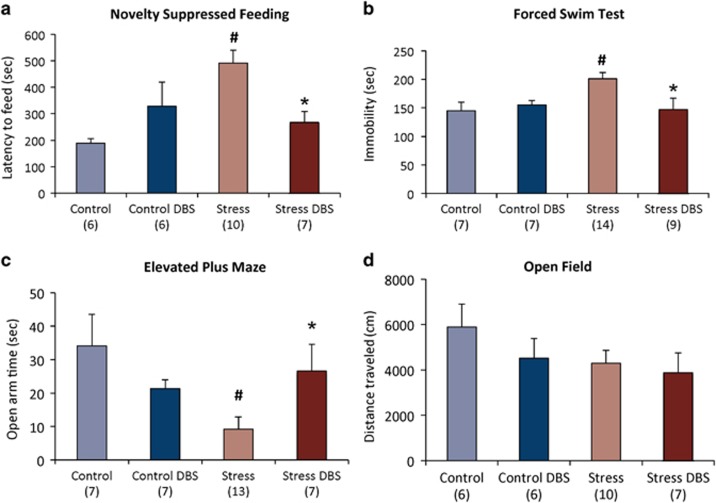
DBS effects in the novelty-suppressed feeding test (NSFT), forced swim test (FST), elevated plus maze test (EPMT) and open field test (OFT). (**a**) In the NSFT, latency to feed was significantly higher in stressed animals as compared with non-stressed controls (*P*=0.0005). DBS applied to stressed animals reduced this latency to near control levels (*P*=0.005). (**b**) In the FST, stressed animals presented significantly more immobility than non-stressed controls (*P*=0.007). DBS applied to stressed animals reduced immobility (*P*=0.006). (**c**) In the EPMT, stressed rats spent significantly less time in the open arms (*P*=0.004 compared with non-stressed controls), an effect that was almost completely reversed by DBS (*P*=0.004). (**d**) Neither stress nor DBS induced significant locomotor changes in the OFT. '#' indicates significant differences between stressed rats and non-stressed controls. '*' indicates significant differences between stressed and Stress+DBS animals. Values are means and s.e. Group sizes are indicated in parenthesis. DBS, deep brain stimulation.

After the first few weeks of CUS, most measures of sucrose preference in stressed animals were significantly lower than in non-stressed controls (*P*<0.01; Figure 1). In addition, stressed animals had a 160% increase in the latency to feed during the NSFT (*P*= 0.0005; Figure 2a), a 39% increase in immobility in the FST (*P*=0.007; Figure 2b) and a 72% decrease in the time spent in the open arms of an EPM (*P*=0.004; Figure 2c). Stressed animals also had a 27% decrease in locomotion in the OF but this difference did not reach statistical significance (Figure 2d). Taken together, these results suggest, to some extent, stress-affected behaviour in virtually every paradigm investigated in our study.

#### DBS effects

In stressed animals receiving DBS, a significant increase in sucrose preference was noticed as early as the second week of treatment (Figure 1; *P*=0.01 as compared with the non-stimulated stressed controls). After 3 weeks of stimulation, sucrose preference in the CUS DBS group was significantly higher than in non-stimulated stressed controls (*P*=0.002; Figure 1). Besides this antianhedonic-like effect, vmPFC stimulation induced anxiolytic- and antidepressant-like responses. When compared with non-stimulated stressed animals, CUS DBS rats had a 45% decrease in the latency to feed in the NSFT (*P*=0.005; Figure 2a), 27% less immobility time in the FST (*P*=0.006; Figure 2b) and spent 188% more time in the open arms of the EPM (*P*=0.004; Figure 2c). In contrast, DBS did not influence stress-induced hypolocomotion in the OF (Figure 2d). The effects of DBS in non-stressed controls were not statistically significant.

### Chronic DBS and neurogenesis

**Figure 3:**
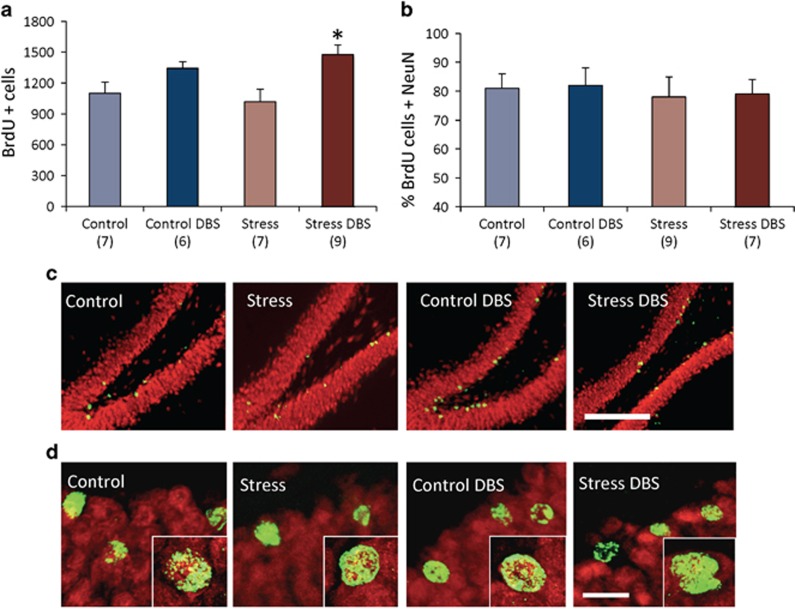
Hippocampal neurogenesis. (**a**) In stressed animals, chronic DBS increased hippocampal neurogenesis by 45% (*P*=0.003). In non-stressed controls, a 22% non-significant increase in the number of BrdU+ cells was seen after DBS. (**b**) Approximately 80–90% of BrdU+ cells co-expressed NeuN, suggesting that these cells assumed a neuronal phenotype. (**c**) Photomicrographs of the hippocampal dentate gyrus illustrating BrdU+ cells (green) dispersed in the granule cell layer (NeuN in red). (**d**) Magnified view (single cells in the right bottom portion of each figure) showing that BrdU+ cells co-stained for NeuN. '*' indicates significant difference between Stress+DBS and non-stimulated stressed animals. Values are means and s.e. Group sizes are indicated in parentheses. Horizontal bar in **c**, 200 μm. Horizontal bar in **d**, 30 μm. BrdU, 5-bromo-2′-deoxyuridine; DBS, deep brain stimulation.

Two-way ANOVA revealed a significant treatment effect (F_(1,27)_=12.52; *P*=0.001) but no stress effect (F_(1,27)_=0.07; *P*=0.8) or a positive stress × DBS interaction (F_(1,27)_=1.21; *P*=0.3) on hippocampal neurogenesis. In stressed animals, chronic vmPFC DBS increased the number of BrdU+ cells by 45% (*P*=0.003; Figure 3a). In non-stressed controls, a 22% non-significant increase in hippocampal neurogenesis was seen after DBS. In all groups, ~80–90% of BrdU+ cells co-expressed NeuN, suggesting that these cells assumed a neuronal phenotype (Figure 3b).

**Figure 4:**
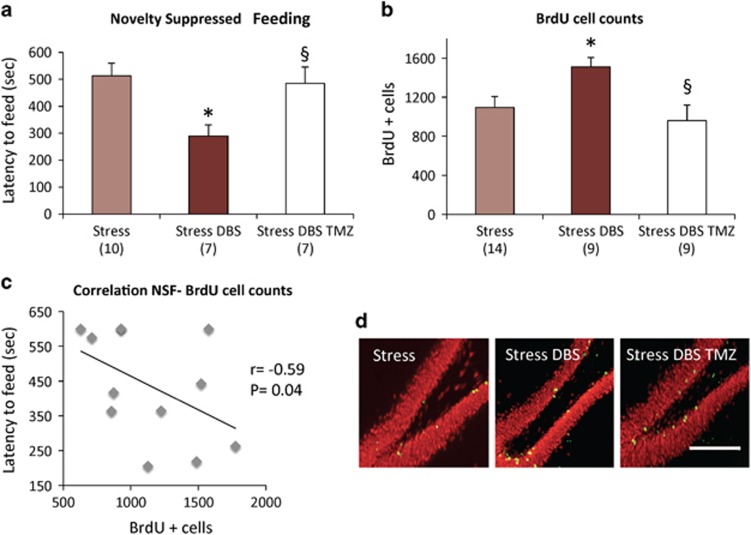
Temozolomide (TMZ), novelty-suppressed feeding and neurogenesis. (**a**) Effect of DBS and DBS TMZ in stressed animals calculated as percentage scores in relation to animals given stress alone. Animals receiving DBS had a significant decrease in the latency to feed, an effect that was practically blocked by TMZ (*P*=0.02). (**b**) Cell counting revealed that TMZ has virtually reversed a stimulation-induced increase in BrdU+ cells in DBS-treated stressed rats (*P*=0.004). (**c**) In addition to group differences in behavioural performance, a significant negative correlation was found between BrdU+ cell counts and latency to feed in the NSFT. (**d**) Photomicrographs of the hippocampal dentate gyrus illustrating BrdU+ cells (green) dispersed in the granule cell layer (NeuN in red). '*' indicates significant difference between Stress+DBS and non-stimulated stressed animals. '§' indicates significant difference between Stress+DBS and Stress+DBS+TMZ. Values are means and s.e.. Group sizes are indicated in parentheses. Horizontal bar in **d**, 200 μm. BrdU, 5-bromo-2′-deoxyuridine; DBS, deep brain stimulation; NSFT, novelty-suppressed feeding test.

**Figure 5:**
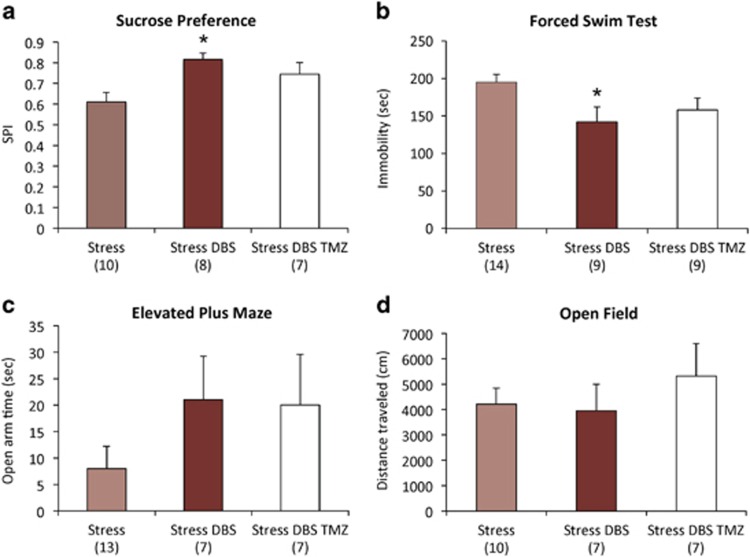
Temozolomide (TMZ) and behaviour. No significant TMZ effects were observed in stressed animals receiving DBS in the (**a**) sucrose preference test, (**b**) forced swim test, (**c**) elevated plus maze or (**d**) open field test. '*' indicates significant difference between Stress+DBS and non-stimulated stressed animals. Values are means and s.e. Group sizes are indicated in parentheses. DBS, deep brain stimulation.

To study whether a DBS-induced increase in neurogenesis could explain its behavioural effects, stressed animals given vmPFC stimulation were injected with TMZ. We found that this agent has only influenced behavioural performance in stressed rats given DBS, having no significant effects in the remainder groups (Supplementary Figures 2–4; Supplementary Tables 2 and 3). The most pronounced consequence of TMZ was to reverse a DBS improvement in performance during the NSFT. Compared with the Stress+DBS alone group, animals receiving Stress+DBS+TMZ had a significant increase in the latency to feed (*P*=0.02; Figure 4a). In contrast, TMZ did not significantly affect DBS-induced improvements in the SPT, FST and EPMT (Figure 5).

Cell counting revealed that TMZ has virtually reversed a stimulation-induced increase in BrdU+ cells in DBS-treated stressed rats (*P*=0.004; Figure 4b). In contrast to behavioural data, however, TMZ has significantly reduced the number of BrdU+ cells in Control and Control+DBS groups, while inducing a non-significant decrease in hippocampal neurogenesis in stressed animals (Supplementary Figure 5). In the Stress+DBS+TMZ group, 82% of cells co-stained for NeuN.

In addition to group differences in behavioural performance, we have conducted correlation analyses to ascertain the potential relationship between neurogenesis and behavioural measures. When all animals undergoing stressed were considered together (that is, including groups receiving TMZ and/or DBS), a significant negative correlation was found between BrdU+ cell counts and latency to feed in the NSFT (*r*=−0.59, *P*=0.04; Figure 4c). In contrast, no significant correlation was found between the number of BrdU+ cells and sucrose preference, time in the open arms of the EPM and immobility time in the FST (Supplementary Table 4).

In summary, our results suggest that the anxiolytic-like response of DBS in stressed animals undergoing the NSFT may be dependent on hippocampal neurogenesis (that is, animals with a higher number of BrdU+ cells have lower anxiety levels). This mechanism, however, does not seem to be responsible for the effects of DBS in the remainder tests.

### BDNF

As stimulation-induced increases in neurogenesis could not explain most DBS effects, we have decided to investigate whether changes in BDNF, an additional substrate involved in chronic antidepressant responses, could be involved. Levels of this neurotrophin were measured in two different brain regions, the prefrontal cortex and hippocampus. In the former, a two-way ANOVA revealed a significant DBS effect (F_(1,18)_=4.61; *P*=0.05) but no effect of stress (F_(1,18)_=0.09; *P*=0.8) or a positive stress × DBS interaction (F_(1,18)_=1.9; *P*=0.2).

**Figure 6:**
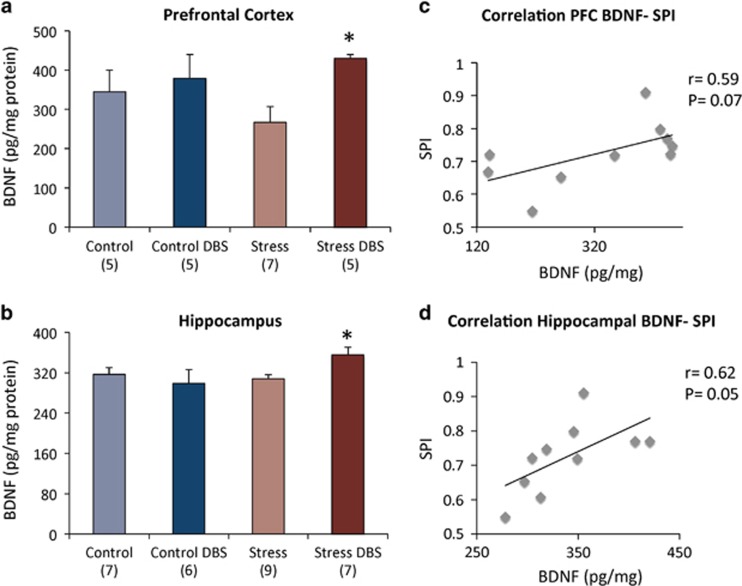
Brain-derived neurotrophic factor (BDNF). Levels of BDNF were measured in two different brain regions, the prefrontal cortex (PFC) and hippocampus. (**a**) In the PFC, a non-significant 22% decrease in BDNF levels was observed when stressed animals were compared with controls. DBS reversed this deficit increasing PFC BDNF by 61% (*P*= 0.02). (**b**) In the hippocampus, BDNF levels were increased by 15% in stressed animals receiving DBS (*P*=0.04). In both PFC (**c**) and hippocampus (**d**), a strong correlation was found between BDNF levels and sucrose preference scores. '*' indicates significant difference between Stress+DBS and non-stimulated stressed animals. Values are means and s.e. Group sizes are indicated in parentheses. DBS, deep brain stimulation.

Though a somewhat similar pattern was observed in the vmPFC and hippocampus, with a lower magnitude of BDNF changes, results did not reach statistical significance (DBS effect F_(1,22)_=0.8, *P*=0.4; Stress effect F_(1,22)_=2.0, *P*=0.2; Interaction F_(1,22)_=3.7, *P*=0.07). In the PFC, a 22% non-significant decrease in BDNF levels was observed when stressed animals were compared with controls (Figure 6a). DBS reversed this deficit increasing PFC BDNF in stressed animals by 61% (*P*=0.02). In the hippocampus, BDNF levels were increased by 15% in stressed animals receiving DBS compared with their non-stimulation stressed mates (*P*=0.04; Figure 6b).

As for neurogenesis, correlation analyses were conducted to establish a potential relationship between neurotrophin levels in stressed animals and behavioural measures in the SPT, NSFT, EPT and FST. A significant positive correlation (*r*= 0.62, *P*= 0.05) and a trend towards significance (*r*= 0.59, *P*= 0.07) were found between sucrose preference scores and hippocampal or PFC BDNF levels, respectively (Figures 6c and d). In contrast, correlations between BDNF levels and latency to feed, time in the open arms of the EPM and immobility time in the FST were not found to be significant (Supplementary Table 4).

In summary, our findings in stressed animals suggest that DBS induced an increase in PFC and hippocampal BDNF and that higher levels of this neurotrophin may be important for the reestablishment of sucrose preference after vmPFC stimulation.

## Discussion

The present results suggest that neuroplasticity-relevant changes, including neurogenesis and increases in BDNF, have a selective role in the beneficial effects of vmPFC stimulation. Overall, chronic DBS exerted robust anxiolytic, antianhedonic- and antidepressant-like activities and reversed various behavioural deficits induced by chronic stress. In parallel, DBS enhanced hippocampal neurogenesis and increased PFC and hippocampal BDNF. TMZ blocked neurogenesis and countered the attenuating action of DBS in the NSFT. Correlation analyses suggest that neuroplastic changes, including neurogenesis and possibly increases in BDNF, may be required for the anxiolytic- and antianhedonic-like effects of DBS but are not crucial for a broader antidepressant-like activity.

### CUS induced behavioural deficits without significantly affecting neurogenesis

CUS-exposed animals had significant reductions in sucrose preference without attenuating neurogenesis. This observation is consistent with those previously reported by Lee *et al.*,^29^ who have shown that stress does not decrease cell proliferation in the dentate gyrus when BrdU is injected after CUS. Similarly, Airan *et al.*^30^ used voltage-sensitive dye imaging to probe hippocampal activity and found that neither CUS was associated with downregulation of hippocampal neurogenesis nor that neurogenesis blockade induces depressive-like activity. In contrast, several reports have shown that CUS may induce decreases in both cell survival and proliferation.^31, 32, 33^ Discrepancies between these results and ours may relate to differences in the type and intensity of stressors; the schedule, frequency and duration of exposures; sex and strain used; and the timing of BrdU administration.^32, 34, 35, 36^ In addition, a number of animal studies have determined that impaired neurogenesis does not consistently precipitate depressive-like behaviour.^37^ With our protocol and BrdU injection timeline, CUS-exposed animals showed clear behavioural deficits but no reduction in neurogenesis.

### CUS exposure induced a broad spectrum of behavioural deficits that were differentially affected by DBS

In addition to an anhedonia-like decline in sucrose preference, stressed animals displayed decreases in sucrose preference and immobility time in the FST and an increase in both latency to feed and time spent in the open arms of an EPM.

In this study, chronic vmPFC DBS followed a protocol previously used by our group.^12^ In contrast to some of our previous reports, however,^10, 11^ we did not include a non-stimulated group (sham) with implanted electrodes. Based on earlier data, sham surgery does not seem to influence hippocampal neurogenesis, BDNF levels and behavioural readouts at long term.^12, 18, 19, 38^ In addition (and in contrast to our previous experiments), sucrose preference scores in the first post-operative week (that is, after the insertion of the electrodes and prior to DBS) were similar to those recorded before the surgery, suggesting that the mere insertion of electrodes into the target did not affect behavioural performance. Finally, the total number of animals used in our study was already elevated. Adding an additional control group with implanted electrodes would have increased this number by ~30–40%.

### TMZ reduced neurogenesis but only blocked the effects of DBS in the NSFT

The antineoplastic drug TMZ has been previously used to block neurogenesis.^21, 24^ We have chosen this agent rather than other neurogenesis ablative treatments (for example radiation, high doses of corticosterone) as it has been shown to decrease the number of BrdU+ cells in the dentate gyrus with minimal transient side effects (for example, leucopenia).^21, 24^ Here, TMZ was injected for 3 days during DBS days 3–5, 2 days prior to BrdU injections. This timeframe was based on previous reports in which rodents given DBS were treated with TMZ and injected with BrdU a few days later.^21^ Newly formed cells are mature and incorporated into the hippocampal circuitry 3–6 weeks after birth.^21, 28^ Our rationale was for TMZ injected a few days after DBS to interfere with cells formed during the first few days of stimulation, that is, neurons that would be mature and functional by the time animals underwent behavioural testing.

Results from our study show that, in stressed animals, TMZ has practically reversed DBS-induced increases in neurogenesis. In behavioural tests, however, TMZ has only blocked the anxiolytic effects of DBS in the NSFT. These findings are in line with previous reports ascribing neurogenesis-dependent and independent mechanisms to conventional antidepressant treatments. Bessa *et al.*^39^ reported that fluoxetine or imipramine retained their ability to reverse CUS-induced behavioural deficits in the SPT and FST when neurogenesis has been blocked by methylazoxymethanol. David *et al.*^3^ found that hippocampal irradiation in mice given corticosterone reversed the anxiolytic effects of fluoxetine in the NSFT without affecting performance in the FST or OFT. Our results are in line with these findings inasmuch as TMZ reduced neurogenesis and reversed DBS effects in the NSFT but not in the FST.

A link between neurogenesis and performance in the NSFT is also supported by the observation that latency to feed in stressed animals was strongly correlated with BrdU cell counts. The possibility that this could have been due to the anxiolytic and neurogenic effects of DBS seems unlikely. As can be seen in Figure 4, correlation was not driven in a few animals only. Our analysis included rats receiving stress alone, Stress+TMZ, Stress+TMZ+DBS and Stress+DBS. While the latter had low latencies to eat, results in the remainder groups were fairly similar. This suggests that the observed correlation was not simply due to a DBS-induced effect on neurogenesis *per se*.

Despite the potential association between neurogenesis and NSFT scores, a few aspects remain unanswered. It is not clear, for example, why CUS-exposed and non-stressed animals had differences in NSFT scores but similar BrdU+ cell counts. In addition, if DBS induces neurogenesis in non-stressed controls, why did these animals not show an improvement in anxiety behaviour? One possibility is that animals in the non-stimulated control group may have reached a ‘floor', below which no improvement in performance is possible. Alternatively, it is conceivable that for the effects of neurogenesis to occur newly borne cells may need to interact with specific substrates that are present or absent after CUS to induce its behavioural effects (such as lower BDNF levels, altered serotonergic transmission and reduced synaptic spines). Future research is needed to clarify these issues.

### BDNF

Previous studies have already suggested a link between increased BDNF and behavioural effects of DBS in CUS-exposed animals.^12, 38^ Here, we found that DBS significantly increased PFC BDNF, having a smaller effect on hippocampal levels of this neurotrophin. The more striking effects of DBS in the PFC suggest a stronger focal effect of this therapy. In addition, a significant negative correlation has been recorded between PFC and hippocampal BDNF levels and sucrose preference scores. This suggests that sucrose preference may be more closely related to BDNF levels, as previously described.^38^

BDNF mediates changes in adult hippocampal neurogenesis and other forms of neuronal plasticity via TrkB (trpomyosin receptor kinase B) receptors.^40^ Overall, this neurotrophin seems to be particularly important for cell proliferation and the differentiation of terminals in newly borne hippocampal neurons.^40, 41^ Mice lacking TrkB receptors have impaired neurogenesis and are behaviourally insensitive to antidepressant treatments.^42^ In our study, stress had an effect on BDNF but did not decrease hippocampal neurogenesis. While DBS increased BDNF and neurogenesis in stressed rats, in non-stressed controls it only affected neurogenesis. This suggests that DBS effects on neurogenesis are likely multifactorial and not solely related to changes in neurotrophin levels.

In summary, our results indicate that DBS exerts multiple effects and counter most behavioural stress responses. Results also show that different behavioural effects may depend on the modulation of distinct substrates. In particular, neuroplastic changes, including neurogenesis and increases in BDNF, may be required for DBS improvements in behaviours with predominant anxiety-like and anhedonic-like components but are unlikely responsible for broader antidepressant-like responses to this therapy.

## Figures and Tables

**Table 1:** Two-way ANOVA comparing behavioural data (DBS and Stress as independent factors)

	*DBS*	*Stress*	*DBS × stress*
NSFT	F_(1,25)_=0.6; *P*=0.4	F_(1,25)_= 4.7; *P*= 0.04	F_(1,25)_= 10.6; *P*= 0.003
FST	F_(1,33)_=2.2; *P*=0.14	F_(1,33)_=2.6; *P*=0.11	F_(1,33)_=4.7; *P*=0.04
EPMT	F_(1,30)_=0.14; *P*=0.7	F_(1,30)_=2.6; *P*=0.1	F_(1,30)_=6.1; *P*=0.02
OFT	F_(1,25)_=1.2; *P*=0.3	F_(1,25)_=1.9; *P*=0.2	F_(1,25)_=0.3; *P*=0.5

	*DBS effect*	*Stress effect*	*Time effect*
SPI^a^	F_(1.26)_=0.2; *P*=0.9	F_(1.26)_=48.2; *P*=0.0001	F_(1.26)_=0.3; *P*=0.8

	*DBS × time*	*Stress × time*	*DBS × Stress × time*
SPI^a^	F_(3,78)_=1.1; *P*=0.3	F_(3,78)_=3.0; *P*=0.04	F_(3,78)_=3.8; *P*=0.014

Abbreviations: ANOVA, analysis of variance; DBS, deep brain stimulation; EPMT, elevated plus maze test; FST, forced swim test; NSFT, novelty-suppressed feeding test; OFT, open field test; SPI, sucrose preference index.

a Sucrose preference scores were calculated with repeated measures ANOVA (DBS, Stress, Time as factors).

## Notes

CH is a consultant for St Jude Medical. The other authors declare no conflict of interest.

## References

[bib1] Santarelli L, Saxe M, Gross C, Surget A, Battaglia F, Dulawa S et al. Requirement of hippocampal neurogenesis for the behavioral effects of antidepressants. Science 2003; 301: 805–809. 12907793 10.1126/science.1083328

[bib2] Duman RS, Monteggia LM. A neurotrophic model for stress-related mood disorders. Biol Psychiatry 2006; 59: 1116–1127. 16631126 10.1016/j.biopsych.2006.02.013

[bib3] David DJ, Samuels BA, Rainer Q, Wang JW, Marsteller D, Mendez I et al. Neurogenesis-dependent and -independent effects of fluoxetine in an animal model of anxiety/depression. Neuron 2009; 62: 479–493. 19477151 10.1016/j.neuron.2009.04.017 PMC2759281

[bib4] Malberg JE, Eisch AJ, Nestler EJ, Duman RS. Chronic antidepressant treatment increases neurogenesis in adult rat hippocampus. J Neurosci 2000; 20: 9104–9110. 11124987 10.1523/JNEUROSCI.20-24-09104.2000 PMC6773038

[bib5] Gould E, Gross CG. Neurogenesis in adult mammals: some progress and problems. J Neurosci 2002; 22: 619–623. 11826089 10.1523/JNEUROSCI.22-03-00619.2002 PMC6758509

[bib6] Nibuya M, Morinobu S, Duman RS. Regulation of BDNF and trkB mRNA in rat brain by chronic electroconvulsive seizure and antidepressant drug treatments. J Neurosci 1995; 15: 7539–7547. 7472505 10.1523/JNEUROSCI.15-11-07539.1995 PMC6578063

[bib7] Holtzheimer PE, Filkowski M, Kelley ME, Gross RE, Mayberg HS. Subcallosal cingulate deep brain stimulation for treatment-resistant unipolar and bipolar depression. Biol Psychiatry 2011; 69: 110S. 10.1001/archgenpsychiatry.2011.1456 PMC4423545 22213770

[bib8] Lozano AM, Mayberg HS, Giacobbe P, Hamani C, Craddock RC, Kennedy SH. Subcallosal cingulate gyrus deep brain stimulation for treatment-resistant depression. Biol Psychiatry 2008; 64: 461–467. 18639234 10.1016/j.biopsych.2008.05.034

[bib9] Mayberg HS, Lozano AM, Voon V, McNeely HE, Seminowicz D, Hamani C et al. Deep brain stimulation for treatment-resistant depression. Neuron 2005; 45: 651–660. 15748841 10.1016/j.neuron.2005.02.014

[bib10] Hamani C, Diwan M, Isabella S, Lozano AM, Nobrega JN. Effects of different stimulation parameters on the antidepressant-like response of medial prefrontal cortex deep brain stimulation in rats. J Psychiatr Res 2010; 44: 683–687. 20096858 10.1016/j.jpsychires.2009.12.010

[bib11] Hamani C, Diwan M, Macedo CE, Brandao ML, Shumake J, Gonzalez-Lima F et al. Antidepressant-like effects of medial prefrontal cortex deep brain stimulation in rats. Biol Psychiatry 2010; 67: 117–124. 19819426 10.1016/j.biopsych.2009.08.025

[bib12] Hamani C, Machado DC, Hipolide DC, Dubiela FP, Suchecki D, Macedo CE et al. Deep brain stimulation reverses anhedonic-like behavior in a chronic model of depression: role of serotonin and brain derived neurotrophic factor. Biol Psychiatry 2012; 71: 30–35. 22000731 10.1016/j.biopsych.2011.08.025 PMC5756076

[bib13] Hamani C, Nobrega JN. Deep brain stimulation in clinical trials and animal models of depression. Eur J Neurosci 2010; 32: 1109–1117. 21039950 10.1111/j.1460-9568.2010.07414.x

[bib14] Hamani C, Nobrega JN. Preclinical studies modeling deep brain stimulation for depression. Biol Psychiatry 2012; 72: 916–923. 22748616 10.1016/j.biopsych.2012.05.024 PMC5633367

[bib15] Hamani C, Temel Y. Deep brain stimulation for psychiatric disease: contributions and validity of animal models. Sci Transl Med 2012; 4: 142rv148. 10.1126/scitranslmed.3003722 22786683

[bib16] McIntyre CC, Savasta M, Walter BL, Vitek JL. How does deep brain stimulation work? Present understanding and future questions. J Clin Neurophysiol 2004; 21: 40–50. 15097293 10.1097/00004691-200401000-00006

[bib17] Florence G, Sameshima K, Fonoff ET, Hamani C. Deep brain stimulation: more complex than the inhibition of cells and excitation of fibers. Neuroscientist 2015; e-pub ahead of print. 10.1177/1073858415591964 26150316

[bib18] Encinas JM, Hamani C, Lozano AM, Enikolopov G. Neurogenic hippocampal targets of deep brain stimulation. J Comp Neurol 2011; 519: 6–20. 21120924 10.1002/cne.22503 PMC3042399

[bib19] Toda H, Hamani C, Fawcett AP, Hutchison WD, Lozano AM. The regulation of adult rodent hippocampal neurogenesis by deep brain stimulation. J Neurosurg 2008; 108: 132–138. 18173322 10.3171/JNS/2008/108/01/0132

[bib20] Hamani C, Stone SS, Garten A, Lozano AM, Winocur G. Memory rescue and enhanced neurogenesis following electrical stimulation of the anterior thalamus in rats treated with corticosterone. Exp Neurol 2011; 232: 100–104. 21906593 10.1016/j.expneurol.2011.08.023

[bib21] Stone SS, Teixeira CM, Devito LM, Zaslavsky K, Josselyn SA, Lozano AM et al. Stimulation of entorhinal cortex promotes adult neurogenesis and facilitates spatial memory. J Neurosci 2011; 31: 13469–13484. 21940440 10.1523/JNEUROSCI.3100-11.2011 PMC6623309

[bib22] Schmuckermair C, Gaburro S, Sah A, Landgraf R, Sartori SB, Singewald N. Behavioral and neurobiological effects of deep brain stimulation in a mouse model of high anxiety- and depression-like behavior. Neuropsychopharmacology 2013; 38: 1234–1244. 23325324 10.1038/npp.2013.21 PMC3656366

[bib23] Liu A, Jain N, Vyas A, Lim LW. Ventromedial prefrontal cortex stimulation enhances memory and hippocampal neurogenesis in the middle-aged rats. eLife 2015; 4: e04803. 10.7554/eLife.04803 PMC4381300 25768425

[bib24] Garthe A, Behr J, Kempermann G. Adult-generated hippocampal neurons allow the flexible use of spatially precise learning strategies. PLoS One 2009; 4: e5464. 19421325 10.1371/journal.pone.0005464 PMC2674212

[bib25] Bambico FR, Nguyen NT, Gobbi G. Decline in serotonergic firing activity and desensitization of 5-HT1A autoreceptors after chronic unpredictable stress. Eur Neuropsychopharmacol 2009; 19: 215–228. 19147333 10.1016/j.euroneuro.2008.11.005

[bib26] Paxinos G, Watson C. The Rat Brain in Stereotaxic Coordinates. Academic Press: San Diego, CA, 1998.

[bib27] Inta D, Lima-Ojeda JM, Lau T, Tang W, Dormann C, Sprengel R et al. Electroconvulsive therapy induces neurogenesis in frontal rat brain areas. PLoS One 2013; 8: e69869. 23922833 10.1371/journal.pone.0069869 PMC3724733

[bib28] Jessberger S, Kempermann G. Adult-born hippocampal neurons mature into activity-dependent responsiveness. Eur J Neurosci 2003; 18: 2707–2712. 14656319 10.1111/j.1460-9568.2003.02986.x

[bib29] Lee KJ, Kim SJ, Kim SW, Choi SH, Shin YC, Park SH et al. Chronic mild stress decreases survival, but not proliferation, of new-born cells in adult rat hippocampus. Exp Mol Med 2006; 38: 44–54. 16520552 10.1038/emm.2006.6

[bib30] Airan RD, Meltzer LA, Roy M, Gong Y, Chen H, Deisseroth K. High-speed imaging reveals neurophysiological links to behavior in an animal model of depression. Science 2007; 317: 819–823. 17615305 10.1126/science.1144400

[bib31] Madeira MD, Cadete-Leite A, Andrade JP, Paula-Barbosa MM. Effects of hypothyroidism upon the granular layer of the dentate gyrus in male and female adult rats: a morphometric study. J Comp Neurol 1991; 314: 171–186. 1797872 10.1002/cne.903140116

[bib32] Pham K, Nacher J, Hof PR, McEwen BS. Repeated restraint stress suppresses neurogenesis and induces biphasic PSA-NCAM expression in the adult rat dentate gyrus. Eur J Neurosci 2003; 17: 879–886. 12603278 10.1046/j.1460-9568.2003.02513.x

[bib33] Heine VM, Maslam S, Zareno J, Joels M, Lucassen PJ. Suppressed proliferation and apoptotic changes in the rat dentate gyrus after acute and chronic stress are reversible. Eur J Neurosci 2004; 19: 131–144. 14750971 10.1046/j.1460-9568.2003.03100.x

[bib34] Gould E, McEwen BS, Tanapat P, Galea LA, Fuchs E. Neurogenesis in the dentate gyrus of the adult tree shrew is regulated by psychosocial stress and NMDA receptor activation. J Neurosci 1997; 17: 2492–2498. 9065509 10.1523/JNEUROSCI.17-07-02492.1997 PMC6573503

[bib35] Tanapat P, Hastings NB, Rydel TA, Galea LA, Gould E. Exposure to fox odor inhibits cell proliferation in the hippocampus of adult rats via an adrenal hormone-dependent mechanism. J Comp Neurol 2001; 437: 496–504. 11503148 10.1002/cne.1297

[bib36] Czeh B, Welt T, Fischer AK, Erhardt A, Schmitt W, Muller MB et al. Chronic psychosocial stress and concomitant repetitive transcranial magnetic stimulation: effects on stress hormone levels and adult hippocampal neurogenesis. Biol Psychiatry 2002; 52: 1057–1065. 12460689 10.1016/s0006-3223(02)01457-9

[bib37] Henn FA, Vollmayr B. Neurogenesis and depression: etiology or epiphenomenon? Biol Psychiatry 2004; 56: 146–150. 15271582 10.1016/j.biopsych.2004.04.011

[bib38] Gersner R, Toth E, Isserles M, Zangen A. Site-specific antidepressant effects of repeated subconvulsive electrical stimulation: potential role of brain-derived neurotrophic factor. Biol Psychiatry 2010; 67: 125–132. 19880094 10.1016/j.biopsych.2009.09.015

[bib39] Bessa JM, Ferreira D, Melo I, Marques F, Cerqueira JJ, Palha JA et al. The mood-improving actions of antidepressants do not depend on neurogenesis but are associated with neuronal remodeling. Mol Psychiatry 2009; 14: 739. 10.1038/mp.2008.119 18982002

[bib40] D'Sa C, Duman RS. Antidepressants and neuroplasticity. Bipolar Disord 2002; 4: 183–194. 12180273 10.1034/j.1399-5618.2002.01203.x

[bib41] Chan JP, Cordeira J, Calderon GA, Iyer LK, Rios M. Depletion of central BDNF in mice impedes terminal differentiation of new granule neurons in the adult hippocampus. Mol Cell Neurosci 2008; 39: 372–383. 18718867 10.1016/j.mcn.2008.07.017 PMC2652348

[bib42] Li Y, Luikart BW, Birnbaum S, Chen J, Kwon CH, Kernie SG et al. TrkB regulates hippocampal neurogenesis and governs sensitivity to antidepressive treatment. Neuron 2008; 59: 399–412. 18701066 10.1016/j.neuron.2008.06.023 PMC2655199

