# Supplementary material for: Neuroplasticity-dependent and -independent mechanisms of chronic deep brain stimulation in stressed rats
Source: Transl Psychiatry. 2015 Nov 3;5(11):e674–. doi: 10.1038/tp.2015.166 (PMC5068759; doi:10.1038/tp.2015.166)
Supplement: Supplementary Table 1 [file tp2015166x1.docx]

**Supplementary Table 1.** Stressors used in the chronic unpredictable stress (CUS) protocol. These were presented at any time during the day or night, singly or in combination, unless otherwise stated to control for stress intensity and duration.

| Stressor | Description | Notes |
| --- | --- | --- |
| 1. Intermittent lighting | Room lights (~350 lx) were turned on and off for 1-2 hours with an interval of 5-30 minutes. |  |
| 1. Light cycle reversal | Light-dark cycle in the vivarium was reversed, and reset after 24 hours. | 24 hours. |
| 1. Stroboscopic illumination | Animals were exposed to stroboscopic lights (5–10 Hz, 2 lx) for 3 to 12 hours in a dim room. |  |
| 1. Food deprivation | Food pellets were removed from the home cage for 12-18 hours. | Not grouped with #10. |
| 1. Water deprivation/empty bottle | Water bottles were removed from the cage lids for 12-18 hours. | Not grouped with #9. |
| 1. Cage tilt | Home cages were tiled at ~45^o^ for 3-12 hours. | Can be overnight or during the day. |
| 1. Used mouse cage | Animals were placed in a used mouse cage for 2 hours. |  |
| 1. Restraint | Animals were held immobile in a plastic restraining cone (Harvard Apparatus) for 30 minutes in the home cage and at room temperature. | Not grouped with #2, 3, 5, 14. |
| 1. Cold restraint | #1 in a 4 ^o^C cold room. | Not grouped with #1, 3, 5, 14. |
| 1. Cold room | Animals were placed in an empty cage at 4^o^C for 2 hours. | Not grouped with #1, 2, 5, 14. |
| 1. Water in cage | Five hundred milliliters of tap water (~10^o^C) was poured in the home cage and removed 12-18 hours later. | Not grouped with #1, 2, 3, 14; overnight only. |
| 1. High frequency sound | Animals were exposed to high frequency noise for 3 hours from an ultrasonic pest repeller (Victor ®). |  |
| 1. Predator odor | One tablespoon of sand with fox urine was placed in the home cage for 3 hours. |  |
| 1. Radio (or static) noise | Animals were exposed to static radio noise (~85 dB) for 3 hours. |  |
| 1. Novel environment | Animals were moved to an unfamiliar room, then placed back to the housing room after 3 hours. |  |
| 1. Forced swim | Animals were forced to swim for 15 min in an inescapable water-filled bin, 20 cm diameter, 50 cm high, 30 cm water depth, 25–27 °C water temperature. | Not grouped with #1, 2, 3, 5. |
| 1. Foreign object exposure | Colored plastic figurine, maximal size of 8 × 8 × 12 cm |  |
| 1. Intraperitoneal injection | Animals received intraperitoneal saline injections with a 25 gauge syringe. |  |
