# Supplementary material for: Neuroplasticity-dependent and -independent mechanisms of chronic deep brain stimulation in stressed rats
Source: Transl Psychiatry. 2015 Nov 3;5(11):e674–. doi: 10.1038/tp.2015.166 (PMC5068759; doi:10.1038/tp.2015.166)
Supplement: Supplementary Table 2 [file tp2015166x2.docx]

**Supplementary Table 2.** Two-way ANOVA comparing behavioural and neurogenetic data in stressed animals given DBS and temozolomide (DBS and TMZ as independent factors).

|  | **DBS** | **TMZ** | **DBS x TMZ** |
| --- | --- | --- | --- |
| **NSFT** | F_(1,29)_=11.2; p=0.002 | F_(1,29)_= 5.2; p= 0.03 | F_(1,29)_= 1.8; p= 0.2 |
| **FST** | F_(1,39)_=4.7; p=0.03 | F_(1,39)_=0.01; p=0.9 | F_(1,39)_=0.6; p=0.04 |
| **EPMT** | F_(1,35)_=1.0; p=0.3 | F_(1,35)_=1.0; p=0.3 | F_(1,35)_=1.2; p=0.3 |
| **OFT** | F_(1,29)_=0.1; p=0.7 | F_(1,29)_=1.7; p=0.2 | F_(1,29)_=0.01; p=0.9 |
| **SPI ^(8th week)^** | F_(1.30)_=6.5; p=0.02 | F_(1.30)_=0.1; p=0.7 | F_(1.30)_=4.1; p=0.05 |
| **BrdU** | F_(1,25)_=7.9; p=0.009 | F_(1,25)_=11.0; p=0.003 | F_(1,25)_=1.0; p=0.3 |

Sucrose preference scores were calculated on the 8^th^ week of stress.

Abbreviations: NSFT = novelty suppressed feeding test; FST = forced swim test; EPMT = elevated plus maze test; OFT = open field test; SPI = sucrose preference index; BrdU= BrdU+ cell count.
