# Supplementary material for: Neuroplasticity-dependent and -independent mechanisms of chronic deep brain stimulation in stressed rats
Source: Transl Psychiatry. 2015 Nov 3;5(11):e674–. doi: 10.1038/tp.2015.166 (PMC5068759; doi:10.1038/tp.2015.166)
Supplement: Supplementary Table 3 [file tp2015166x3.docx]

**Supplementary Table 3.** Two-way ANOVA comparing behavioural and neurogenetic data in non-stressed animals receiving DBS and temozolomide (DBS and TMZ as independent factors).

|  | **DBS** | **TMZ** | **DBS x TMZ** |
| --- | --- | --- | --- |
| **NSFT** | F_(1,20)_=1.9; p=0.2 | F_(1,20)_= 1.8; p= 0.2 | F_(1,20)_= 0.2; p= 0.7 |
| **FST** | F_(1,23)_=0.3; p=0.5 | F_(1,23)_=3.8; p=0.06 | F_(1,23)_=0.02; p=0.6 |
| **EPMT** | F_(1,21)_=0.6; p=0.4 | F_(1,21)_=0.5; p=0.5 | F_(1,21)_=0.6; p=0.4 |
| **OFT** | F_(1,18)_=3.0; p=0.1 | F_(1,18)_=2.2; p=0.1 | F_(1,18)_=0.8; p=0.4 |
| **SPI ^(8th week)^** | F_(1.30)_=0.03; p=0.9 | F_(1.30)_=0.08; p=0.7 | F_(1.30)_=3.8; p=0.06 |
| **BrdU** | F_(1,25)_=13.4; p=0.001 | F_(1,25)_=14.4; p=0.0009 | F_(1,25)_=1.1; p=0.3 |

Sucrose preference scores were calculated on the 8^th^ week of stress.

Abbreviations: NSFT = novelty suppressed feeding test; FST = forced swim test; EPMT = elevated plus maze test; OFT = open field test; SPI = sucrose preference index; BrdU= BrdU+ cell count.
