# Supplementary material for: Neuroplasticity-dependent and -independent mechanisms of chronic deep brain stimulation in stressed rats
Source: Transl Psychiatry. 2015 Nov 3;5(11):e674–. doi: 10.1038/tp.2015.166 (PMC5068759; doi:10.1038/tp.2015.166)
Supplement: Supplementary Table 4 [file tp2015166x4.docx]

**Supplementary Table 4.** Correlation between BrdU cell counts or BDNF levels and behavioural performance.

|  | **SPT**  Sucrose preference | **NSFT**  Latency to feed | **EPMT**  Time in open arms | **FST**  Immobility time |
| --- | --- | --- | --- | --- |
| BrdU+ cells  Stressed animals | 0.26  (-0.27 to 0.69) | -0.59  (-0.87 to -0.03) | 0.39  (-0.49 to 0.52) | -0.18  (-0.64 to 0.36) |
| PFC BDNF  Stressed animals | 0.59  (-0.13 to 0.89) | 0.10  (-0.6 to 0.7) | 0.25  (-0.41 to 0.74) | -0.15  (-0.74 to 0.57) |
| Hipp BDNF  Stressed animals | 0.62  (0.01 to 0.89) | 0.02  (-0.65 to 0.67) | 0.03  (-0.56 to 0.59) | -0.14  (-0.64 to 0.44) |

BDNF- brain derived neurotrophic factor; EPMT- elevated plus maze test; FST- forced swim test; Hipp- Hippocampus; NSFT- novelty suppressed feeding test; PFC- prefrontal cortex; SPT- sucrose preference test. Values represent Pearson coefficient (95% confidence intervals).
