# Supplementary figures and images for: Neuroplasticity-dependent and -independent mechanisms of chronic deep brain stimulation in stressed rats
Source: Transl Psychiatry. 2015 Nov 3;5(11):e674–. doi: 10.1038/tp.2015.166 (PMC5068759; doi:10.1038/tp.2015.166)

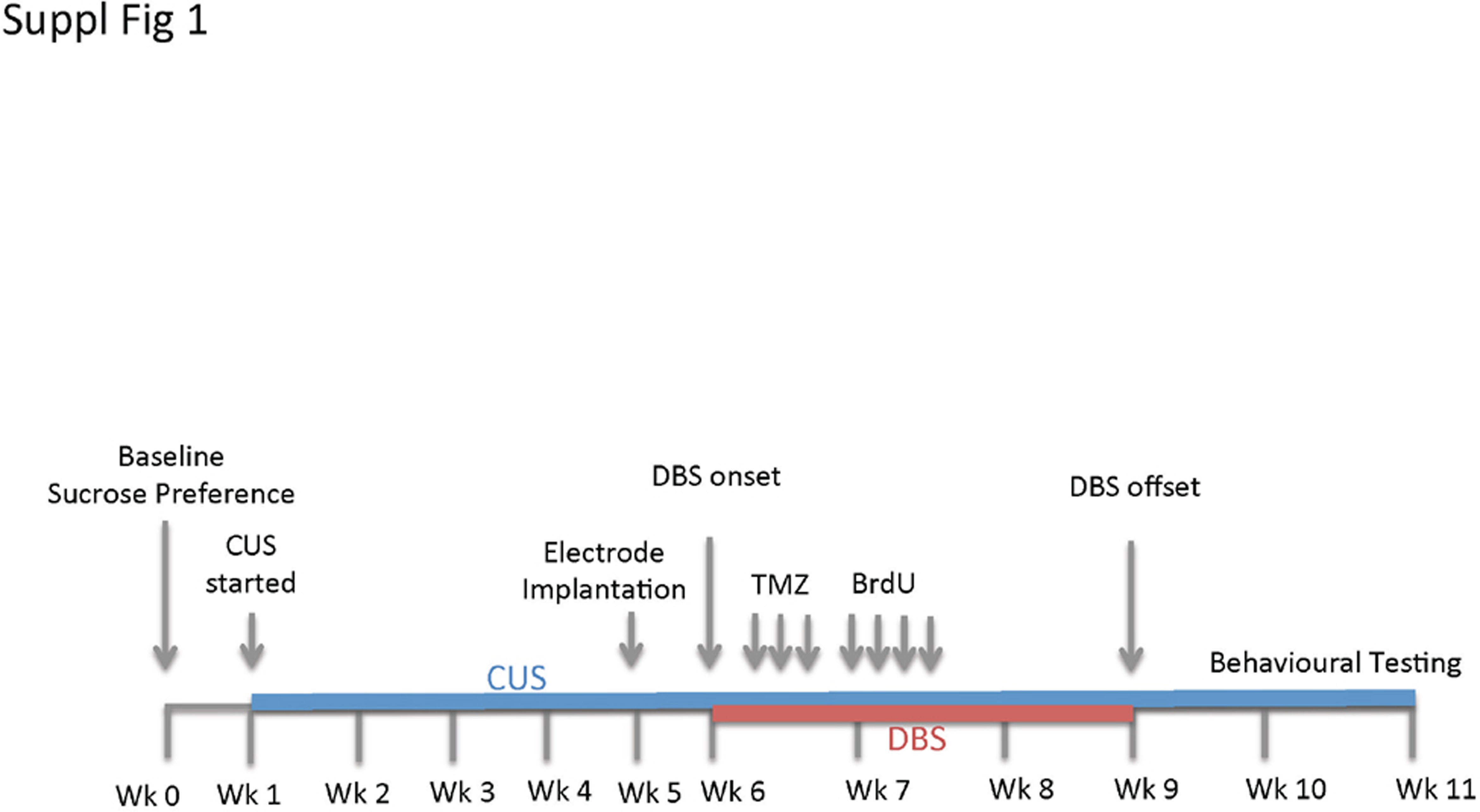

Supplement: Supplementary Figure 1 [file tp2015166x5.tif]

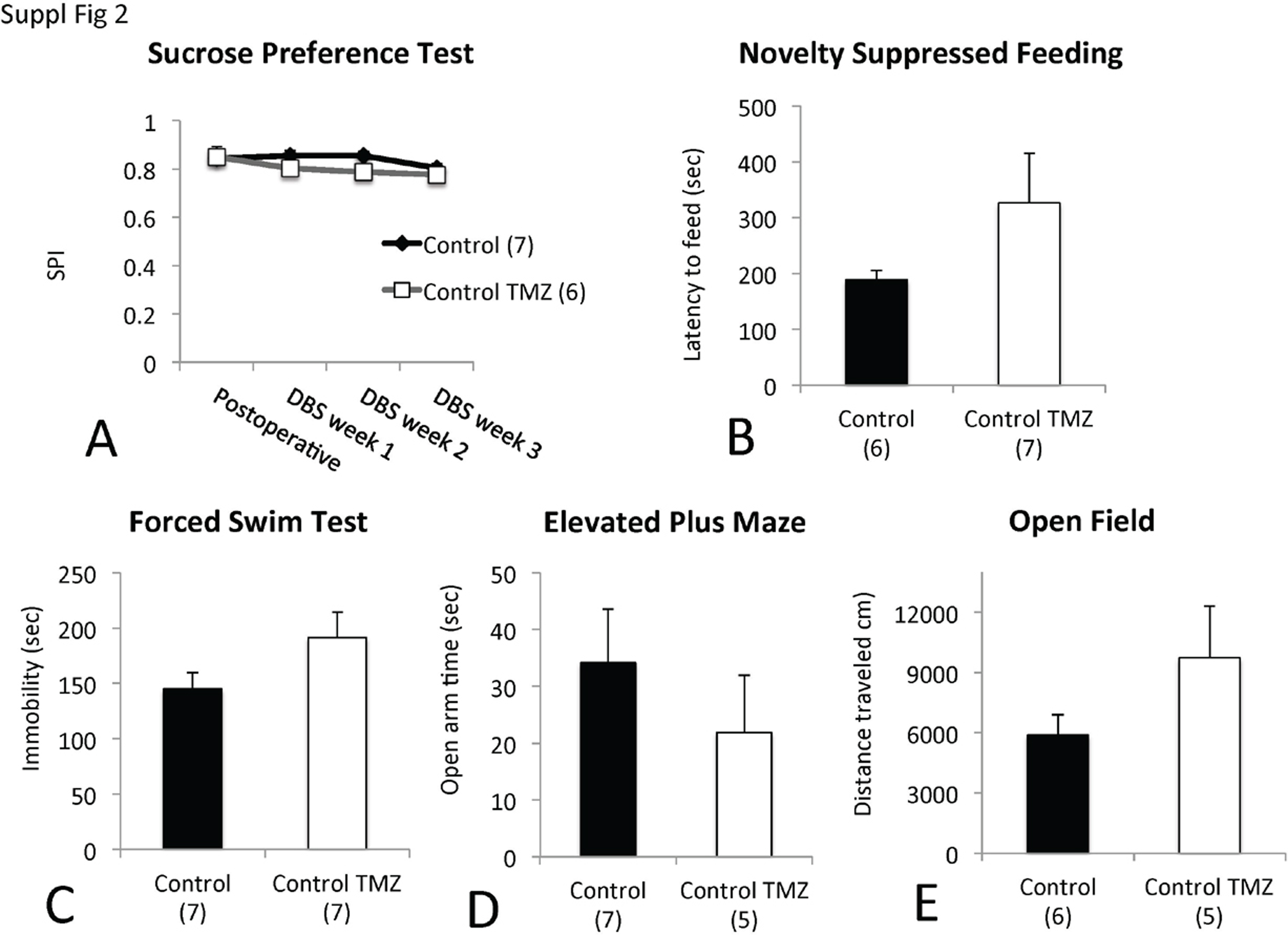

Supplement: Supplementary Figure 2 [file tp2015166x6.tif]

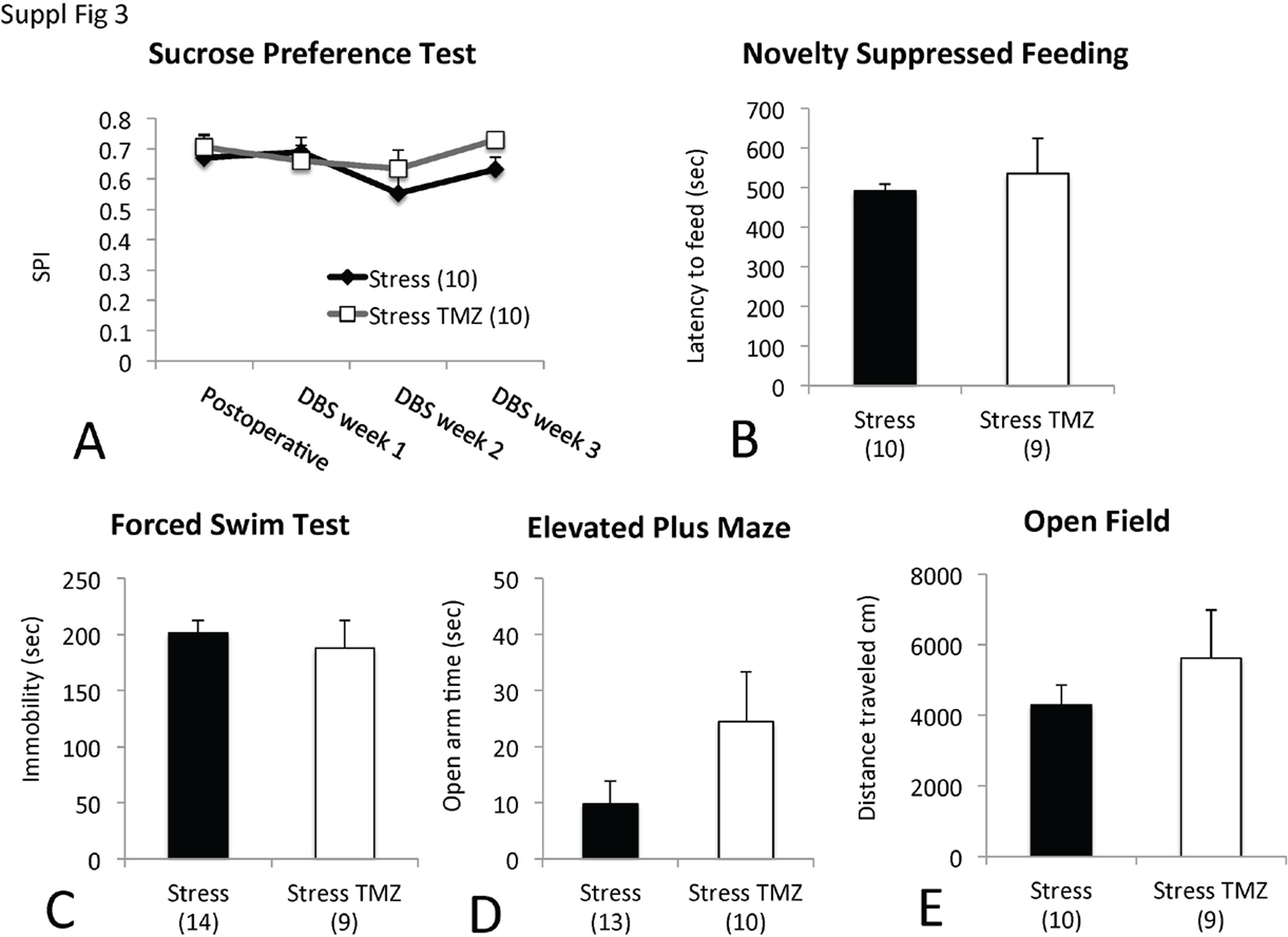

Supplement: Supplementary Figure 3 [file tp2015166x7.tif]

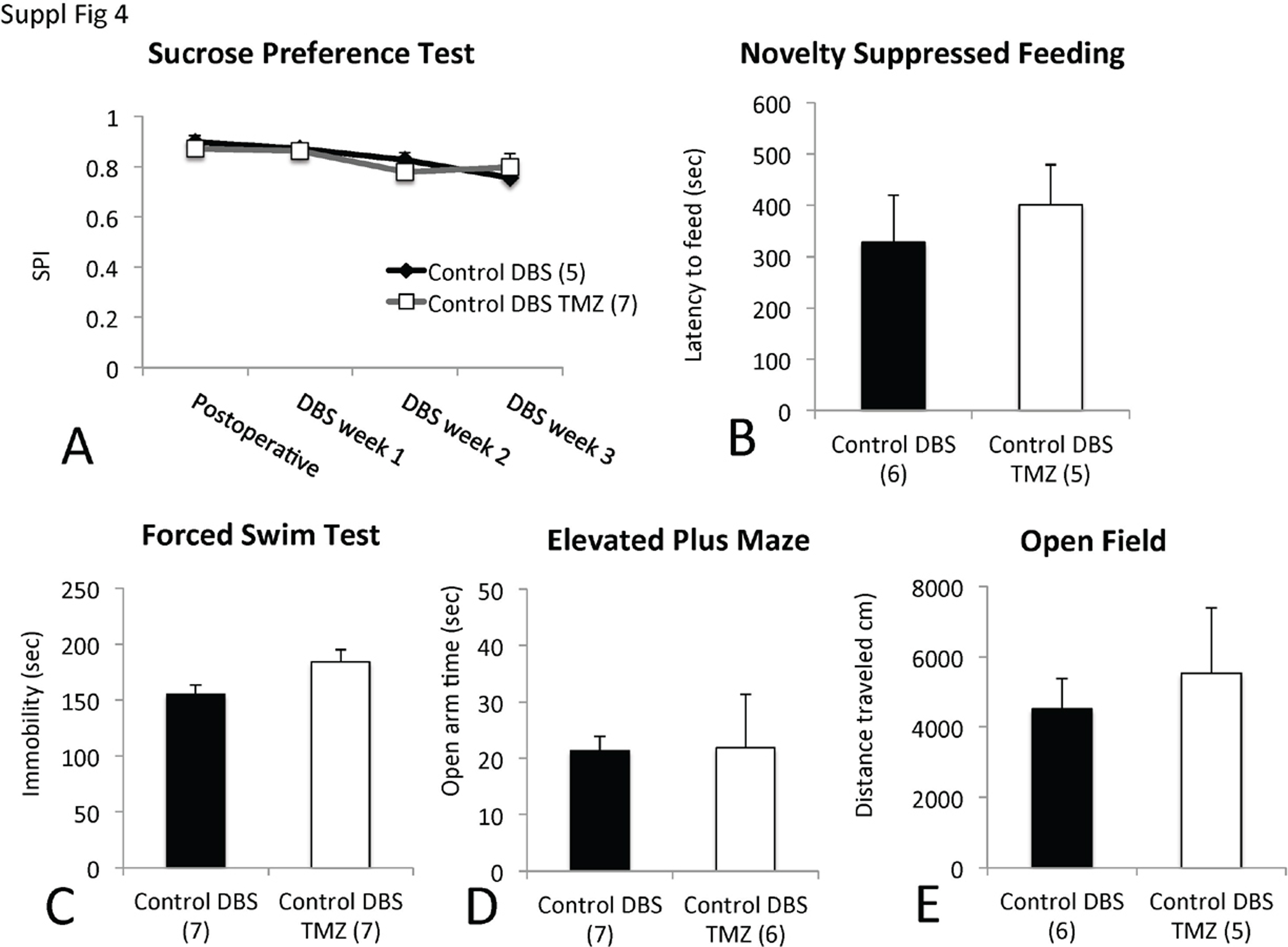

Supplement: Supplementary Figure 4 [file tp2015166x8.tif]

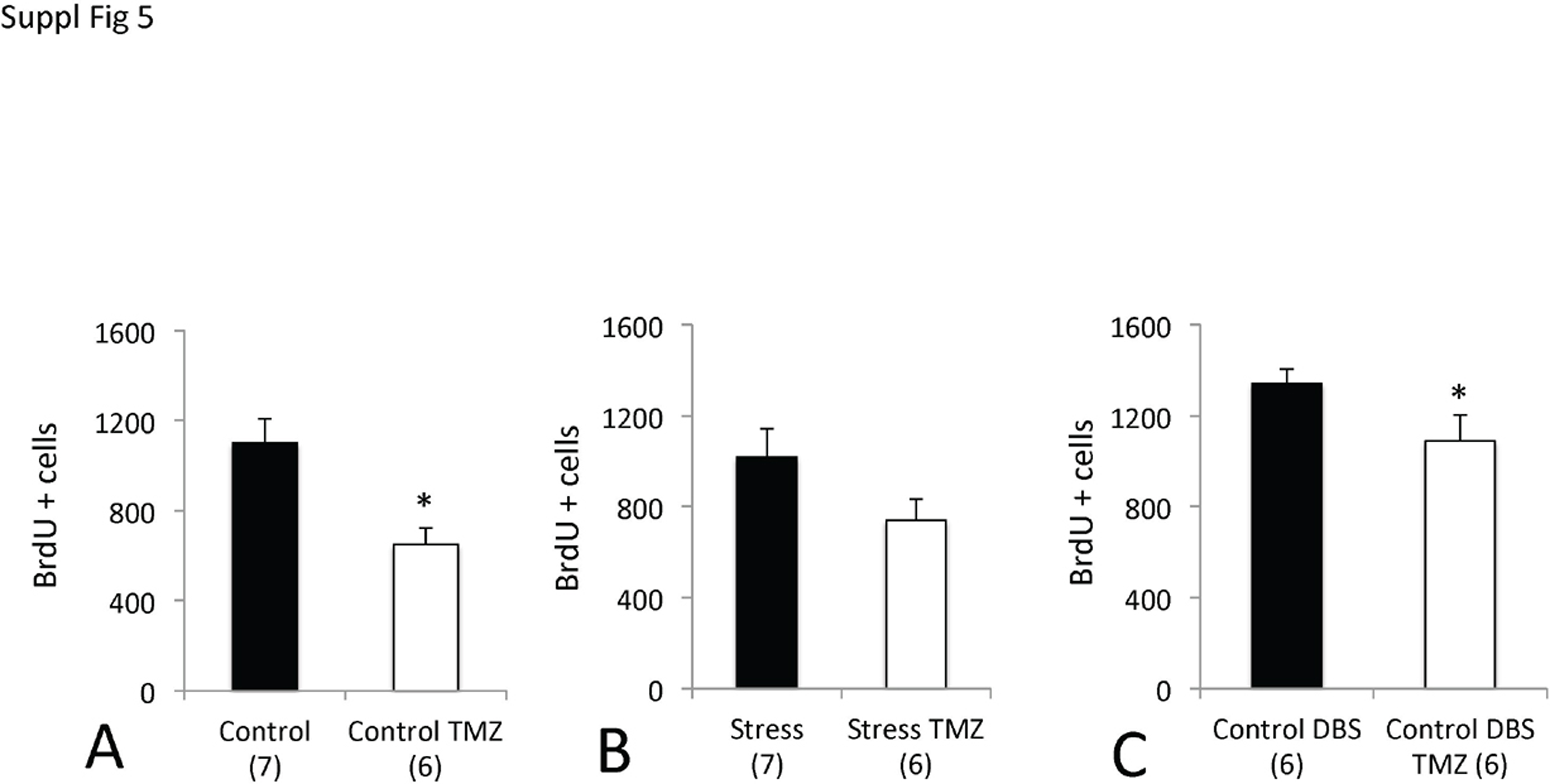

Supplement: Supplementary Figure 5 [file tp2015166x9.tif]
