# Supplementary material for: Neuroplasticity-dependent and -independent mechanisms of chronic deep brain stimulation in stressed rats
Source: Transl Psychiatry. 2015 Nov 3;5(11):e674–. doi: 10.1038/tp.2015.166 (PMC5068759; doi:10.1038/tp.2015.166)
Supplement: Supplementary Information [file tp2015166x10.docx]

**Supplementary Figure 1.** Timeline of the experiments. Fisher rats underwent chronic unpredictable mild stress for four consecutive weeks. Preference for drinking a sucrose solution was measured on a weekly basis. On the fifth week following stress, animals underwent electrode implantation. One week later, deep brain stimulation (DBS) was conducted for 3 consecutive weeks (from weeks 6-8; 8h/day). After stimulation was discontinued, rats were tested in multiple behavioural paradigms. BrdU was injected from stimulation days 7-10 (50mg/Kg twice/day). Temozolomide (TMZ) was administered from stimulation days 3-5 (50mg/Kg/day). BrdU=5-bromo- 2’deoxyuridine; CUS= chronic unpredictable mild stress; DBS= deep brain stimulation; SP= sucrose preference; wk= week.

**Supplementary Figure 2.** Behavioural effects of temozolomide in non-stressed controls. No significant differences across groups were found in the (A) sucrose preference test, (B) novelty suppressed feeding, (C), forced swim test, (D) elevated plus maze, and (E) open field test. Values are means and standard errors. Group sizes are indicated in parentheses.

**Supplementary Figure 3.** Behavioural effects of temozolomide in stressed rats. No significant differences across groups were found in the (A) sucrose preference test, (B) novelty suppressed feeding, (C), forced swim test, (D) elevated plus maze, and (E) open field test. Values are means and standard errors. Group sizes are indicated in parentheses.

**Supplementary Figure 4.** Behavioural effects of temozolomide in non-stressed controls given DBS. No significant differences across groups were found in the (A) sucrose preference test, (B) novelty suppressed feeding, (C), forced swim test, (D) elevated plus maze, and (E) open field test. Values are means and standard errors. Group sizes are indicated in parentheses.

**Supplementary Figure 5** Neurogenesis and temozolomide (TMZ). TMZ has significantly reduced the number of BrdU+ cells in (A) controls (p=0.001) but not in (B) stressed rats. In control animals receiving DBS (C), TMZ has countered the effects of stimulation (p=0.004). * significant differences between TMZ treated animals and their respective groups of interest. Values are means and standard errors. Group sizes are indicated in parentheses.
